# Impact & Blast Traumatic Brain Injury: Implications for Therapy

**DOI:** 10.3390/molecules23020245

**Published:** 2018-01-26

**Authors:** Satoshi Yamamoto, Douglas S. DeWitt, Donald S. Prough

**Affiliations:** Department of Anesthesiology, University of Texas Medical Branch, Galveston, TX 77555, USA; ddewitt@utmb.edu (D.S.D.); dsprough@utmb.edu (D.S.P.)

**Keywords:** traumatic brain injury, therapeutic strategy, brain trauma foundation guideline

## Abstract

Traumatic brain injury (TBI) is one of the most frequent causes of combat casualties in Operations Iraqi Freedom (OIF), Enduring Freedom (OEF), and New Dawn (OND). Although less common than combat-related blast exposure, there have been significant numbers of blast injuries in civilian populations in the United States. Current United States Department of Defense (DoD) ICD-9 derived diagnoses of TBI in the DoD Health Care System show that, for 2016, severe and moderate TBIs accounted for just 0.7% and 12.9%, respectively, of the total of 13,634 brain injuries, while mild TBIs (mTBIs) accounted for 86% of the total. Although there is a report that there are differences in the frequency of long-term complications in mTBI between blast and non-blast TBIs, clinical presentation is classified by severity score rather than mechanism because severity scoring is associated with prognosis in clinical practice. Blast TBI (bTBI) is unique in its pathology and mechanism, but there is no treatment specific for bTBIs—these patients are treated similarly to TBIs in general and therapy is tailored on an individual basis. Currently there is no neuroprotective drug recommended by the clinical guidelines based on evidence.

## 1. Introduction

Traumatic brain injury (TBI) is one of the most frequent causes of combat casualties in Operations Iraqi Freedom (OIF), Enduring Freedom (OEF), and New Dawn (OND) [1,2,3,4]. As many as 23% of warfighters returning from the conflicts in Iraq and Afghanistan sustained a TBI [5,6] and at least 60% of these TBIs are due to blast exposure [7,8]. According to Department of Defense statistics from 2015, blast exposure is the main cause of combat casualties with 73% of combat injuries in U.S. forces due to blasts [9,10]. Although less common than combat-related blast exposure, there have been significant numbers of blast injuries in civilian populations in the U.S. [11]. Kapur et al., reported 5931 injuries and 699 deaths due to blasts in the U.S. from 1983 to 2002 [12].

In this article, we review the current understanding of blast TBIs (bTBIs), contrasting with traditional TBIs, and feature common approaches to clinical management to highlight the guidelines for TBIs in general.

## 2. Epidemiology

### 2.1. Blast TBI

According to reports from OIF and OEF (Operations Iraqi and Enduring Freedom), the patterns of combat-related injuries have shifted from penetration injuries to blastrelated injuries. Since 4 November 2006, blasts have been the most common cause of injury among American soldiers [13]. Through to March of 2004, 97% of the casualties suffered by the First Light Armored Reconnaissance Battalion of the First Marine Division were related to blast [14]. Of the approximately one million veterans screened for TBI between 2007–2015, 8.4% were characterized as having sustained a TBI; most were mild and the majority due to blast exposure. Since an additional 45,000 had a previous diagnosis of mTBI, a total of 137,841 (13.8%) of those screened had sustained a combat-related TBI [15]. One striking feature of the mTBI cases seen in veterans of the wars in Iraq and Afghanistan is the high association between mTBI and posttraumatic stress disorder [2]. Approximately 75% of non-combat TBIs (e.g., motor vehicle accidents, sports-related TBI, etc.) sustained by U.S. military personnel annually are classified as mild [16]. Between 2000 and 2016, 82.3% of TBIs among military personnel were mild [17,18] and approximately 80% of mTBIs were due to blast [19].

### 2.2. TBI in General

Approximately 1.74 million people sustain a TBI in the United States each year [20]. As injuries to the brain are been classified as “head and neck” injuries [21], it might be difficult to know the precise ratio of TBIs that occur. Current United States Department of Defense (DoD) ICD-9 derived diagnoses of TBI in the DoD Health Care System show that, for 2016, severe and moderate TBI accounted for just 0.7% and 12.9%, respectively, of the total of 13,634 brain injuries, while mTBI brain injury accounted for 86% of the total [18].

The prognosis for an appropriately managed mTBI patient is good, generally resulting in complete recovery [22,23]. Those who have experienced mTBI usually fully recover within a year. Nevertheless, there can be a variety of short- and long-term sequelae of mTBIs in some patients [24,25,26,27,28]. Potential chronic effects of mTBI include post-concussion syndrome [23,29], post-traumatic headaches [30,31,32], post-traumatic epilepsy [33], post-traumatic vertigo [34,35], and chronic traumatic encephalopathy [29,36,37,38].

Moderate and severe TBIs are associated with lingering neurological deficits and functional impairments [28], and only one fourth of survivors of severe TBIs are able to achieve long-term functional independence [39,40,41]. The prevalence of long-term disability related to severe TBIs is reported to be approximately 1–2% of the population in the United States [42,43].

While a myriad of outcome prediction models designed to guide clinical decision-making have been developed, the complexity of severe head injury makes it difficult to apply many of these models to clinical decision-making in individual TBI patients [44,45,46].

## 3. Blast Physics

Blast processes involve the energy propagation of an explosive source into the surrounding environment, followed by interaction, loading, and damage of materials, structures, and systems. In biological systems, the extreme shifts of blast wave over (positive) pressure and under (negative) pressure of explosions brings substantial cellular disruption due to the stresses imparted by those rapidly changing positive and negative pressures [28].

In blast TBI, the blast wave encountering the head reflects and diffracts, resulting in highly transient and spatially non-uniform loading over the skull. High reflected pressures appear on the surface facing the incident wave. The combination of head size, skull thickness, and skull elasticity may exaggerate biomechanical responses to blast-wave loading [47].

## 4. Mechanism and Pathophysiology

### 4.1. Blast TBI

In most respects, blast TBI has clinical aspects of closed head injury. Primary blast injuries are defined as injuries that are a direct result of blast wave-induced over and under pressures. Secondary blast injury is due to impact from objects (e.g., shrapnel) put in motion by the blast. Tertiary blast injury is due to the acceleration of the body by the blast wind, and quaternary blast injury is any other type of injury (e.g., burns, lung damage from toxic gases, etc.) [48,49].

Although the exact mechanisms through which primary blast exposure causes tissue injury are uncertain, the most commonly stated are spallation, implosion, inertial effects [50,51,52], and cavitation [53,54]. Spallation occurs when the shock wave passes from a dense to a less dense material, causing the more dense material to fragment into the less dense material [52]. A relatively simple example is an explosion under water, causing the more dense water to spall (spray) into the less dense air [52]. Spallation may contribute to primary blast-induced lung injury [55]. Cavitation and implosion are related phenomena that occur as the negative pressure phase causes dissolved gases to form bubbles in fluids [54,56]. These bubbles are compressed (imploded) by the negative pressure and then expand explosively as the negative pressure phase passes [53,54]. Cavitation and implosion also have been implicated in the pathophysiology of pulmonary primary blast injury [55]. Inertial forces occur at the boundaries of tissues with different densities when the blast pressure accelerates materials of different densities at different rates, thereby creating shearing forces [52]. To date, there is no direct experimental evidence implicating any of these mechanisms in the pathophysiology of bTBI. However, studies using computerized and physical models suggest that cavitation may contribute to tissue injury secondary to blast exposure [54]. Furthermore, finite element modeling studies indicate that cerebrospinal fluid (CSF) cavitation could occur at the pressures and durations encountered in real-world blast events [57,58]. Generally, these effects occur primarily at the junction between tissues of different densities, which is consistent with observations of prominent astroglial scarring at grey–white matter junctions and in structures lining the ventricles [59]. Although primary blast overpressure may be a factor that contributes to brain injury and cognitive dysfunction, especially if amplified by the proximity of vehicles, buildings, and other solid structures, the threshold for bTBI remains to be known [28].

Some blast-exposed patients have evidence of TBI in the absence of trauma. These indirect blast effects may be precipitated by the transmission of blast energies through thoracic and abdominal blood vessels, as well as vagally-mediated bradycardia, arterial hypotension, and perhaps the resulting cerebral hypoperfusion [28]. There are two types of theoretical proposals for this mechanism. One is blast wave propagation directly through the skull or sinus openings [60]. Another is thoracoabdominal compression that transmits blast pressures to cerebral vascular and cerebrospinal fluid systems [61]. The integrity of the blood–brain barrier is impaired by increased cerebral vascular pressure, leading to the damage of small cerebral vessels [62]. As noted above, blast-induced damage to air-filled organs, such as the lungs, can create air emboli through the process of spallation [63]. The emboli may travel to the cerebral vasculature, leading to cerebral ischemia and infarction [49]. Also, exposure to blast pressures leads to arterial remodeling that may be associated with blast-induced vasospasm [64].

### 4.2. TBI in General

TBI itself is divided into two separate, but related categories: primary brain injury and secondary brain injury.

Primary brain injury occurs at the time of trauma, i.e., damage resulting directly from external mechanical forces transferred to intracranial contents. These include a combination of focal contusions and hematomas, as well as shearing of white matter tracts (diffuse axonal injury) along with cerebral edema and swelling [65,66]. Shearing mechanisms lead to diffuse axonal injury (DAI), which is visualized pathologically and on neuroimaging studies as multiple small lesions seen within white matter tracts.

Focal cerebral contusions are the most frequently encountered lesions. Contusions are commonly seen in the basal frontal and temporal areas, which are particularly susceptible due to direct impact on basal skull surfaces in the setting of acceleration/deceleration injuries.

Extra-axial hematomas are, in general, encountered when forces are distributed to the cranial vault and the most superficial cerebral layers. These are seen as epidural, subdural, and subarachnoid hemorrhage.

Secondary brain injury results from a cascade of molecular mechanisms that are initiated at the time of the first contact and sustained for hours or days. These mechanisms include neurotransmitter-mediated excitotoxicity from glutamate release, free-radical injury to cell membranes, electrolyte imbalances, mitochondrial dysfunction, inflammatory responses, apoptosis, secondary ischemia from vasospasm, focal microvascular occlusion, and vascular injury [41,59,67,68,69,70,71,72]. The mechanisms involved in cell death and tissue loss after TBI include complex, multifaceted interactions between acute and delayed biochemical, molecular, physiological, and anatomical events. In the affected brain region with high oxidative stress, free iron released from hemoglobin contributes to the production of free radicals [73]. Nitrosylation of proteins, another indicator of oxidative/nitrative stress, is also markedly increased [28]. The specific combination and magnitude of secondary mechanisms may vary with the actual biomechanics of the initial injury process.

## 5. Clinical Presentation and Classification

Although there is a report of differences in the frequency of long-term complications in mTBIs between blast and non-blast TBIs [74], the preponderance of evidence indicates that the neurological, psychological, and behavioral consequences of blast and non-blast TBIs are similar [17,75,76,77].

Regardless of the biomechanical causes of injury, it is important to make a careful, accurate initial diagnosis, especially with mTBI patients who may be unable to recall what happened at the at the time of injury due to alterations in consciousness. Immediately after TBI, patients often exhibit loss of consciousness, memory loss, headache, confusion, nausea, and focal neurologic deficits. In the long term, patients with TBI report cognitive impairment and neuropsychological symptoms (behavior and personality changes, depression, and suicidality), Parkinsonism, and other speech and gait abnormalities [29,36,78,79].

TBI has traditionally been classified using injury severity scores, the most common of which is the Glasgow Coma Scale (GCS) [80] (Table 1). The GCS is universally accepted as a tool for TBI classification due to its simplicity, reproducibility, and predictive value for overall prognosis. A GCS score of 13 to 15 is considered mild injury, 9 to 12 is considered moderate injury, and 8 or less as severe traumatic brain injury. However, for practical clinical use, the severity of initial impairment after TBI is subdivided into two major categories: mild TBI and moderate/severe TBI. The Marshall scale (Table 2) and the Rotterdam scale (Table 3) are two currently used CT-based grading scales [65,81].

Mild TBI is defined by loss or alteration of consciousness for up to 30 min after injury, a confused or disoriented state lasting less than 24 h, normal structural brain imaging on computerized tomographic (CT) scanning, and a Glasgow Coma Scale score of 13–15.

Moderate/severe TBI is defined by a traumatically-induced physiological disruption of brain function as manifested by either loss of consciousness for greater than 30 min, an initial GCS of 12 or less after 30 min, or post-traumatic amnesia for greater than 24 h.

## 6. Management

As there is no treatment specific for bTBI, these patients are treated similarly to TBI in general and therapy is tailored on an individual basis. For mild TBI, the mainstay of treatment is rest and targeted treatment of clinical symptoms. Observation is recommended for at least 24 h after a mild TBI [82,83]. Hospital admission is recommended for patients at risk for such immediate complications from head injury as GCS < 15, abnormal CT findings, seizures, and comorbid coagulopathy [84,85,86,87]. Attention has been focused on mTBI recently because of the putative relationship between repeated mTBI and the early onset of dementias such as Alzheimer’s in retired athletes, especially professional football players [88]. The numbers of sports-related concussions has been estimated to be as high 300,000 annually in the United States [89]. Based on the assumption that the 300,000 mTBIs involving loss of consciousness represented only 8% [90] and 19.2% [91] of sports-related TBIs, Langlois et al. estimated that the actual number of TBIs in contact sports may range from 1.6 to 3.8 million [92]. The considerable range of the estimates of the number of sport-related TBIs (i.e., 50,000 to 3.8 million) emphasizes the importance of accurate definitions of and/or criteria for mTBI.

Surgical treatment is indicated based on neurological status and head CT result criteria for moderate/severe TBI. The initial treatment of moderate/severe TBI should follow Adult Trauma Life Support (ATLS) guidelines [93] as to take into consideration systemic stabilization. Anesthesiologists play a critical role in securing airways, as well as obtaining large bore intravenous or central lines for cardiopulmonary resuscitation, in addition to placing arterial lines to closely monitor hemodynamics. Since patients with moderate/severe TBI often wear neck collars for cervical spine stabilization, fiberoptic intubation may be required. Moreover, in cases requiring placement of a central line in the internal jugular vein, stabilization of the neck with assistance is vital. Although hypotension and hypoxia should be avoided, hyperoxia with a PaO_2_ > 300 mmHg in patients with severe TBI is associated with higher in-hospital fatalities [94].

For moderate/severe TBIs without surgical indications, the goal of treatment is to limit the likelihood of secondary posttraumatic hypotension and hypoxia, both of which markedly increase mortality and morbidity [95,96], by maintaining BP (systolic > 90 mmHg) and oxygenation (PaO_2_ > 60 mmHg) for TBI patients in the ICU. In general, patients with TBI should be monitored closely to maintain euvolemia. Electrolyte disturbances are commonly seen in patients with TBI and should be assessed on a regular basis with other labs.

Although there has been some debate about the specifics of treatment and monitoring for patients with TBI, the Brain Trauma Foundation recently published revised 4th edition guidelines for severe TBI, which is endorsed by neurosurgical professional organizations [97]. The guidelines have been reviewed by high quality studies [95].

Currently, there are no evidence-based recommendations for the use of neuroprotective agents in TBI patients. However, there have been some clinical studies that show the effectiveness of neuroprotective agents in TBI [98,99,100,101,102], but none of these studies is of sufficiently high quality to warrant the use of neuroprotective agents.

## 7. Conclusions

Although blast TBIs appear to be unique in terms of pathology and biomechanical injury mechanisms, bTBI patients are indistinguishable from non-bTBI patients [17,48,75,76,77]. Although standards of care for TBI patients have been developed and refined over the last 20 years with several revisions of national and international guidelines, solid evidence is scarce. Clinical decisions should be made case-by-case based on current standard of care guidelines.

## Figures and Tables

**Table 1:** Glasgow Coma Scale (GCS). A GCS score of 13 to 15 is considered mild injury, 9 to 12 is considered moderate injury, and 8 or less as severe traumatic brain injury.

Response	Score
Eye opening	
Spontaneous	4
Response to verbal command	3
Response to pain	2
No eye opening	1
Best verbal response	
Oriented	5
Confused	4
Inappropriate words	3
Incomprehensible sounds	2
No verbal response	1
Best motor response	
Obeys commands	6
Localizing response to pain	5
Withdrawal response to pain	4
Flexion to pain	3
Extension to pain	2
No motor response	1

**Table 2:** Marshall CT score of traumatic brain injury.

Category	Definition
Diffuse injury I (no visible pathology)	No visible intracranial pathology seen on CT scan
Diffuse injury II	Cisterns are present with midline shift of 0–5 mm and/or lesions densities present; no high or mixed density lesion >25 cm^3^ may include bone fragments and foreign bodies
Diffuse injury III (swelling)	Cisterns compressed or absent with midline shift 0–5 mm; no high or mixed density lesion >25 cm^3^
Diffuse injury IV (shift)	Midline shift >5 mm; no high or mixed density lesion >25 cm^3^
Evacuated mass lesion V	Any lesion surgically evacuated
Non-evacuated mass lesion VI	High or mixed density lesion >25 cm^3^; not surgically evacuated

**Table 3:** Rotterdam CT score. In adults, mortality at six months increases with the score; score 1: 0%, score 2: 7%, score 3: 16%, score 4: 26%, score 5: 53%, score 6: 61%.

Predictor Value	Score
Basal cisterns	
Normal	0
Compressed	1
Absent	2
Midline shift	
No shift or shift ≤ 5 mm	0
Shift > 5 mm	1
Epidural mass lesion	
Present	0
Absent	1
Intraventricular blood or subarachnoid hemorrhage	
Absent	0
Present	1

## References

[1] Cernak I., Noble-Haeusslein L.J. (2010). Traumatic brain injury: An overview of pathobiology with emphasis on military populations. J. Cereb. Blood Flow Metab..

[2] Elder G.A., Cristian A. (2009). Blast-related mild traumatic brain injury: Mechanisms of injury and impact on clinical care. Mt. Sinai J. Med. J. Transl. Pers. Med..

[3] Eskridge S.L., Macera C.A., Galarneau M.R., Holbrook T.L., Woodruff S.I., MacGregor A.J., Morton D.J., Shaffer R.A. (2012). Injuries from combat explosions in Iraq: Injury type, location, and severity. Injury.

[4] MacGregor A.J., Dougherty A.L., Morrison R.H., Quinn K.H., Galarneau M.R. (2011). Repeated concussion among U.S. military personnel during Operation Iraqi Freedom. J. Rehabil. Res. Dev..

[5] Tanielian T., Haycox L.H., Schell T.L., Marshall G.N., Burnam M.A., Eibner C., Karney B.R., Meredith L.S., Ringel J.S., Vaiana M.E. (2008). Invisible Wounds of War: Summary and Recommendations for Addressing Psychological and Cognitive Injuries.

[6] Terrio H., Brenner L.A., Ivins B.J., Cho J.M., Helmick K., Schwab K., Scally K., Bretthauer R., Warden D. (2009). Traumatic brain injury screening: Preliminary findings in a US Army Brigade Combat Team. J. Head Trauma Rehabil..

[7] Bell M.J., Kochanek P.M. (2008). Traumatic brain injury in children: Recent advances in management. Indian J. Pediatr..

[8] McKee A.C., Daneshvar D.H., Alvarez V.E., Stein T.D. (2014). The neuropathology of sport. Acta Neuropathol. (Berl.).

[9] Kocsis J.D., Tessler A. (2009). Pathology of blast-related brain injury. J. Rehabil. Res. Dev..

[10] Long J.B., Bentley T.L., Wessner K.A., Cerone C., Sweeney S., Bauman R.A. (2009). Blast overpressure in rats: Recreating a battlefield injury in the laboratory. J. Neurotrauma.

[11] McDonald J.S., Brinjikji W., Rabinstein A.A., Cloft H.J., Lanzino G., Kallmes D.F. (2015). Conscious sedation versus general anaesthesia during mechanical thrombectomy for stroke: A propensity score analysis. J. Neurointerv. Surg..

[12] Kapur G.B., Hutson H.R., Davis M.A., Rice P.L. (2005). The United States twenty-year experience with bombing incidents: Implications for terrorism preparedness and medical response. J. Trauma.

[13] Wilk J.E., Thomas J.L., McGurk D.M., Riviere L.A., Castro C.A., Hoge C.W. (2010). Mild traumatic brain injury (concussion) during combat: Lack of association of blast mechanism with persistent postconcussive symptoms. J. Head Trauma Rehabil..

[14] Gondusky J.S., Reiter M.P. (2005). Protecting military convoys in Iraq: An examination of battle injuries sustained by a mechanized battalion during Operation Iraqi Freedom II. Mil. Med..

[15] DePalma R.G., Hoffman S.W. (2016). Combat blast related traumatic brain injury (TBI): Decade of recognition; promise of progress. Behav. Brain Res..

[16] Baldassarre M., Smith B., Harp J., Herrold A., High W.M., Babcock-Parziale J., Louise-Bender Pape T. (2015). Exploring the Relationship Between Mild Traumatic Brain Injury Exposure and the Presence and Severity of Postconcussive Symptoms Among Veterans Deployed to Iraq and Afghanistan. PM R.

[17] Mac Donald C.L., Johnson A.M., Wierzechowski L., Kassner E., Stewart T., Nelson E.C., Werner N.J., Zonies D., Oh J., Fang R. (2014). Prospectively assessed clinical outcomes in concussive blast vs nonblast traumatic brain injury among evacuated US military personnel. JAMA Neurol..

[18] DoD Worldwide Numbers for TBI. http://dvbic.dcoe.mil/dod-worldwide-numbers-tbi.

[19] Mishra V., Skotak M., Schuetz H., Heller A., Haorah J., Chandra N. (2016). Primary blast causes mild, moderate, severe and lethal TBI with increasing blast overpressures: Experimental rat injury model. Sci. Rep..

[20] Traumatic Brain Injury|Concussion|Traumatic Brain Injury|CDC Injury Center. https://www.cdc.gov/TraumaticBrainInjury/index.html.

[21] Xydakis M.S., Fravell M.D., Nasser K.E., Casler J.D. (2005). Analysis of Battlefield Head and Neck Injuries in Iraq and Afghanistan. Otolaryngol. Head Neck Surg..

[22] Evans R.W. (1992). The postconcussion syndrome and the sequelae of mild head injury. Neurol. Clin..

[23] Kushner D. (1998). Mild traumatic brain injury: Toward understanding manifestations and treatment. Arch. Intern. Med..

[24] Kors E.E., Terwindt G.M., Vermeulen F.L., Fitzsimons R.B., Jardine P.E., Heywood P., Love S., van den Maagdenberg A.M., Haan J., Frants R.R. (2001). Delayed cerebral edema and fatal coma after minor head trauma: Role of the CACNA1A calcium channel subunit gene and relationship with familial hemiplegic migraine. Ann. Neurol..

[25] Haas D.C., Lourie H. (1984). Delayed deterioration of consciousness after trivial head injury in childhood. Br. Med. J. Clin. Res. Ed..

[26] Wetjen N.M., Pichelmann M.A., Atkinson J.L.D. (2010). Second impact syndrome: Concussion and second injury brain complications. J. Am. Coll. Surg..

[27] Jordan B.D. (2013). The clinical spectrum of sport-related traumatic brain injury. Nat. Rev. Neurol..

[28] Masel B.E., DeWitt D.S. (2010). Traumatic brain injury: A disease process, not an event. J. Neurotrauma.

[29] Dikmen S.S., Corrigan J.D., Levin H.S., Machamer J., Stiers W., Weisskopf M.G. (2009). Cognitive outcome following traumatic brain injury. J. Head Trauma Rehabil..

[30] Baandrup L., Jensen R. (2005). Chronic post-traumatic headache--a clinical analysis in relation to the International Headache Classification 2nd Edition. Cephalalgia Int. J. Headache.

[31] Paniak C., Reynolds S., Phillips K., Toller-Lobe G., Melnyk A., Nagy J. (2002). Patient complaints within 1 month of mild traumatic brain injury: A controlled study. Arch. Clin. Neuropsychol..

[32] Nampiaparampil D.E. (2008). Prevalence of chronic pain after traumatic brain injury: A systematic review. JAMA.

[33] Annegers J.F., Grabow J.D., Groover R.V., Laws E.R., Elveback L.R., Kurland L.T. (1980). Seizures after head trauma: A population study. Neurology.

[34] Friedman J.M. (2004). Post-traumatic vertigo. Med. Health Rhode Island.

[35] Hoffer M.E., Gottshall K.R., Moore R., Balough B.J., Wester D. (2004). Characterizing and treating dizziness after mild head trauma. Otol. Neurotol..

[36] McAllister T.W., Flashman L.A., Maerlender A., Greenwald R.M., Beckwith J.G., Tosteson T.D., Crisco J.J., Brolinson P.G., Duma S.M., Duhaime A.-C. (2012). Cognitive effects of one season of head impacts in a cohort of collegiate contact sport athletes. Neurology.

[37] Gronwall D., Wrightson P. (1975). Cumulative effect of concussion. Lancet Lond. Engl..

[38] Covassin T., Elbin R., Kontos A., Larson E. (2010). Investigating baseline neurocognitive performance between male and female athletes with a history of multiple concussion. J. Neurol. Neurosurg. Psychiatry.

[39] Schreiber M.A., Aoki N., Scott B.G., Beck J.R. (2002). Determinants of mortality in patients with severe blunt head injury. Arch. Surg..

[40] Jiang J.-Y., Gao G.-Y., Li W.-P., Yu M.-K., Zhu C. (2002). Early indicators of prognosis in 846 cases of severe traumatic brain injury. J. Neurotrauma.

[41] Rosner M.J., Rosner S.D., Johnson A.H. (1995). Cerebral perfusion pressure: Management protocol and clinical results. J. Neurosurg..

[42] Thurman D.J., Alverson C., Dunn K.A., Guerrero J., Sniezek J.E. (1999). Traumatic brain injury in the United States: A public health perspective. J. Head Trauma Rehabil..

[43] Zaloshnja E., Miller T., Langlois J.A., Selassie A.W. (2008). Prevalence of long-term disability from traumatic brain injury in the civilian population of the United States, 2005. J. Head Trauma Rehabil..

[44] Lingsma H.F., Roozenbeek B., Steyerberg E.W., Murray G.D., Maas A.I.R. (2010). Early prognosis in traumatic brain injury: From prophecies to predictions. Lancet Neurol..

[45] Yuan F., Ding J., Chen H., Guo Y., Wang G., Gao W.-W., Chen S.-W., Tian H.-L. (2012). Predicting outcomes after traumatic brain injury: The development and validation of prognostic models based on admission characteristics. J. Trauma Acute Care Surg..

[46] Roozenbeek B., Lingsma H.F., Lecky F.E., Lu J., Weir J., Butcher I., McHugh G.S., Murray G.D., Perel P., Maas A.I. (2012). International Mission on Prognosis Analysis of Clinical Trials in Traumatic Brain Injury (IMPACT) Study Group; Corticosteroid Randomisation After Significant Head Injury (CRASH) Trial Collaborators; Trauma Audit and Research Network (TARN) Prediction of outcome after moderate and severe traumatic brain injury: External validation of the International Mission on Prognosis and Analysis of Clinical Trials (IMPACT) and Corticoid Randomisation after Significant Head injury (CRASH) prognostic models. Crit. Care Med..

[47] Bolander R. (2012). A Multi-Species Analysis of Biomechanical Responses of the Head to a Shock Wave.

[48] Cooper G.J., Maynard R.L., Cross N.L., Hill J.F. (1983). Casualties from terrorist bombings. J. Trauma.

[49] Mayorga M.A. (1997). The pathology of primary blast overpressure injury. Toxicology.

[50] Schardin H. (1950). The physical principles of the effects of a detonation. Ger. Aviat. Med. World War II.

[51] Stuhmiller J.H., Phillips Y.Y., Richmond D.R. (1991). The Physics and Mechanisms of Primary Blast Injury: Conventional Warfare: Ballistic, Blast, and Burn Injuries.

[52] Wolf S.J., Bebarta V.S., Bonnett C.J., Pons P.T., Cantrill S.V. (2009). Blast injuries. Lancet Lond. Engl..

[53] Nakagawa A., Manley G.T., Gean A.D., Ohtani K., Armonda R., Tsukamoto A., Yamamoto H., Takayama K., Tominaga T. (2011). Mechanisms of primary blast-induced traumatic brain injury: Insights from shock-wave research. J. Neurotrauma.

[54] Goeller J., Wardlaw A., Treichler D., O’Bruba J., Weiss G. (2012). Investigation of cavitation as a possible damage mechanism in blast-induced traumatic brain injury. J. Neurotrauma.

[55] Ho A.M.-H. (2002). A simple conceptual model of primary pulmonary blast injury. Med. Hypotheses.

[56] Young R.P., Collins D.S. (1999). Monitoring an experimental tunnel seal in granite using acoustic emission and ultrasonic velocity. Rock Mech. Ind..

[57] Panzer M.B., Myers B.S., Capehart B.P., Bass C.R. (2012). Development of a finite element model for blast brain injury and the effects of CSF cavitation. Ann. Biomed. Eng..

[58] Bass C.R., Panzer M.B., Rafaels K.A., Wood G., Shridharani J., Capehart B. (2012). Brain injuries from blast. Ann. Biomed. Eng..

[59] Yi J.-H., Hazell A.S. (2006). Excitotoxic mechanisms and the role of astrocytic glutamate transporters in traumatic brain injury. Neurochem. Int..

[60] Säljö A., Arrhén F., Bolouri H., Mayorga M., Hamberger A. (2008). Neuropathology and pressure in the pig brain resulting from low-impulse noise exposure. J. Neurotrauma.

[61] Gupta R.K., Przekwas A. (2013). Mathematical Models of Blast-Induced TBI: Current Status, Challenges, and Prospects. Front. Neurol..

[62] Chodobski A., Zink B.J., Szmydynger-Chodobska J. (2011). Blood-brain barrier pathophysiology in traumatic brain injury. Transl. Stroke Res..

[63] Committee on Gulf War and Health: Long-Term Effects of Blast Exposures, Board on the Health of Select Populations, Institute of Medicine (2014). Pathophysiology of Blast Injury and Overview of Experimental Data.

[64] Alford P.W., Dabiri B.E., Goss J.A., Hemphill M.A., Brigham M.D., Parker K.K. (2011). Blast-induced phenotypic switching in cerebral vasospasm. Proc. Natl. Acad. Sci. USA.

[65] Maas A.I.R., Hukkelhoven C.W.P.M., Marshall L.F., Steyerberg E.W. (2005). Prediction of outcome in traumatic brain injury with computed tomographic characteristics: A comparison between the computed tomographic classification and combinations of computed tomographic predictors. Neurosurgery.

[66] Moen K.G., Skandsen T., Folvik M., Brezova V., Kvistad K.A., Rydland J., Manley G.T., Vik A. (2012). A longitudinal MRI study of traumatic axonal injury in patients with moderate and severe traumatic brain injury. J. Neurol. Neurosurg. Psychiatry.

[67] Coles J.P., Minhas P.S., Fryer T.D., Smielewski P., Aigbirihio F., Donovan T., Downey S.P.M.J., Williams G., Chatfield D., Matthews J.C. (2002). Effect of hyperventilation on cerebral blood flow in traumatic head injury: Clinical relevance and monitoring correlates. Crit. Care Med..

[68] Büki A., Koizumi H., Povlishock J.T. (1999). Moderate posttraumatic hypothermia decreases early calpain-mediated proteolysis and concomitant cytoskeletal compromise in traumatic axonal injury. Exp. Neurol..

[69] Diringer M.N., Videen T.O., Yundt K., Zazulia A.R., Aiyagari V., Dacey R.G., Grubb R.L., Powers W.J. (2002). Regional cerebrovascular and metabolic effects of hyperventilation after severe traumatic brain injury. J. Neurosurg..

[70] Morganti-Kossmann M.C., Rancan M., Stahel P.F., Kossmann T. (2002). Inflammatory response in acute traumatic brain injury: A double-edged sword. Curr. Opin. Crit. Care.

[71] Oertel M., Boscardin W.J., Obrist W.D., Glenn T.C., McArthur D.L., Gravori T., Lee J.H., Martin N.A. (2005). Posttraumatic vasospasm: The epidemiology, severity, and time course of an underestimated phenomenon: A prospective study performed in 299 patients. J. Neurosurg..

[72] Vespa P., Bergsneider M., Hattori N., Wu H.-M., Huang S.-C., Martin N.A., Glenn T.C., McArthur D.L., Hovda D.A. (2005). Metabolic crisis without brain ischemia is common after traumatic brain injury: a combined microdialysis and positron emission tomography study. J. Cereb. Blood Flow Metab. Off. J. Int. Soc. Cereb. Blood Flow Metab..

[73] Chang E.F., Claus C.P., Vreman H.J., Wong R.J., Noble-Haeusslein L.J. (2005). Heme regulation in traumatic brain injury: Relevance to the adult and developing brain. J. Cereb. Blood Flow Metab..

[74] Luethcke C.A., Bryan C.J., Morrow C.E., Isler W.C. (2010). Comparison of concussive symptoms, cognitive performance, and psychological symptoms between acute blast-versus nonblast-induced mild traumatic brain injury. J. Int. Neuropsychol. Soc..

[75] Belanger H.G., Proctor-Weber Z., Kretzmer T., Kim M., French L.M., Vanderploeg R.D. (2011). Symptom complaints following reports of blast versus non-blast mild TBI: Does mechanism of injury matter?. Clin. Neuropsychol..

[76] Dretsch M.N., Kelly M.P., Coldren R.L., Parish R.V., Russell M.L. (2015). No Significant Acute and Subacute Differences between Blast and Blunt Concussions across Multiple Neurocognitive Measures and Symptoms in Deployed Soldiers. J. Neurotrauma.

[77] Lange R.T., Pancholi S., Brickell T.A., Sakura S., Bhagwat A., Merritt V., French L.M. (2012). Neuropsychological outcome from blast versus non-blast: Mild traumatic brain injury in U.S. military service members. J. Int. Neuropsychol. Soc. JINS.

[78] DeKosky S.T., Blennow K., Ikonomovic M.D., Gandy S. (2013). Acute and chronic traumatic encephalopathies: Pathogenesis and biomarkers. Nat. Rev. Neurol..

[79] Wang H.-K., Lin S.-H., Sung P.-S., Wu M.-H., Hung K.-W., Wang L.-C., Huang C.-Y., Lu K., Chen H.-J., Tsai K.-J. (2012). Population based study on patients with traumatic brain injury suggests increased risk of dementia. J. Neurol. Neurosurg. Psychiatry.

[80] Teasdale G., Jennett B. (1974). Assessment of coma and impaired consciousness. A practical scale. Lancet Lond. Engl..

[81] Marshall L.F., Marshall S.B., Klauber M.R., Van Berkum Clark M., Eisenberg H., Jane J.A., Luerssen T.G., Marmarou A., Foulkes M.A. (1992). The diagnosis of head injury requires a classification based on computed axial tomography. J. Neurotrauma.

[82] American Academy of Pediatrics (1999). The management of Minor Closed head injury in children. Pediatrics.

[83] Lawler K.A., Terrigino C.A. (1996). Guidelines for evaluation and education of adult patients with mild traumatic brain injuries in an acute care hospital setting. J. Head Trauma Rehabil..

[84] Stein S.C., Ross S.E. (1990). The value of computed tomographic scans in patients with low-risk head injuries. Neurosurgery.

[85] Dacey R.G., Alves W.M., Rimel R.W., Winn H.R., Jane J.A. (1986). Neurosurgical complications after apparently minor head injury. Assessment of risk in a series of 610 patients. J. Neurosurg..

[86] Atzema C., Mower W.R., Hoffman J.R., Holmes J.F., Killian A.J., Oman J.A., Shen A.H., Greenwood S.D., National Emergency X-Radiography Utilization Study (NEXUS) II Group (2004). Defining “therapeutically inconsequential” head computed tomographic findings in patients with blunt head trauma. Ann. Emerg. Med..

[87] Nishijima D.K., Offerman S.R., Ballard D.W., Vinson D.R., Chettipally U.K., Rauchwerger A.S., Reed M.E., Holmes J.F. (2012). Clinical Research in Emergency Services and Treatment (CREST) Network Immediate and delayed traumatic intracranial hemorrhage in patients with head trauma and preinjury warfarin or clopidogrel use. Ann. Emerg. Med..

[88] Schwartz I., Tsenter J., Shochina M., Shiri S., Kedary M., Katz-Leurer M., Meiner Z. (2007). Rehabilitation outcomes of terror victims with multiple traumas. Arch. Phys. Med. Rehabil..

[89] Thurman D.J., Branche C.M., Sniezek J.E. (1998). The epidemiology of sports-related traumatic brain injuries in the United States: Recent developments. J. Head Trauma Rehabil..

[90] Schulz M.R., Marshall S.W., Mueller F.O., Yang J., Weaver N.L., Kalsbeek W.D., Bowling J.M. (2004). Incidence and risk factors for concussion in high school athletes, North Carolina, 1996–1999. Am. J. Epidemiol..

[91] Collins M.W., Hawn K.L. (2002). The clinical management of sports concussion. Curr. Sports Med. Rep..

[92] Langlois J.A., Rutland-Brown W., Wald M.M. (2006). The epidemiology and impact of traumatic brain injury: A brief overview. J. Head Trauma Rehabil..

[93] Advanced Trauma Life Support. https://www.facs.org/quality-programs/trauma/atls.

[94] Rincon F., Kang J., Vibbert M., Urtecho J., Athar M.K., Jallo J. (2014). Significance of arterial hyperoxia and relationship with case fatality in traumatic brain injury: A multicentre cohort study. J. Neurol. Neurosurg. Psychiatry.

[95] Brain Trauma Foundation. https://braintrauma.org/guidelines/guidelines-for-the-management-of-severe-tbi-4th-ed#/.

[96] Chesnut R.M., Marshall L.F., Klauber M.R., Blunt B.A., Baldwin N., Eisenberg H.M., Jane J.A., Marmarou A., Foulkes M.A. (1993). The role of secondary brain injury in determining outcome from severe head injury. J. Trauma.

[97] Guidelines for the Management of Severe Traumatic Brain Injury 4th Edition. https://braintrauma.org/uploads/03/12/Guidelines_for_Management_of_Severe_TBI_4th_Edition.pdf.

[98] Xiao G., Wei J., Wu Z., Wang W., Jiang Q., Cheng J., Lu F., Wu J., Xu H., Fang R. (2007). Clinical study on the therapeutic effects and mechanism of progesterone in the treatment for acute severe head injury. Zhonghua Wai Ke Za Zhi.

[99] Wright D.W., Kellermann A.L., Hertzberg V.S., Clark P.L., Frankel M., Goldstein F.C., Salomone J.P., Dent L.L., Harris O.A., Ander D.S. (2007). ProTECT: A randomized clinical trial of progesterone for acute traumatic brain injury. Ann. Emerg. Med..

[100] Razmkon A., Sadidi A., Sherafat-Kazemzadeh E., Mehrafshan A., Jamali M., Malekpour B., Saghafinia M. (2011). Administration of vitamin C and vitamin E in severe head injury: A randomized double-blind controlled trial. Clin. Neurosurg..

[101] Aminmansour B., Nikbakht H., Ghorbani A., Rezvani M., Rahmani P., Torkashvand M., Nourian M., Moradi M. (2012). Comparison of the administration of progesterone versus progesterone and vitamin D in improvement of outcomes in patients with traumatic brain injury: A randomized clinical trial with placebo group. Adv. Biomed. Res..

[102] Hoffer M.E., Balaban C., Slade M.D., Tsao J.W., Hoffer B. (2013). Amelioration of acute sequelae of blast induced mild traumatic brain injury by *N*-acetyl cysteine: A double-blind, placebo controlled study. PLoS ONE.

